# Transcatheter Aortic Valve Implantation in Alkaptonuria-Аssociated Severe Aortic Stenosis: A 2.5-Year Follow-Up Case Report and Literature Review

**DOI:** 10.3390/life15050737

**Published:** 2025-05-02

**Authors:** Spas Kitov, Maria-Florance Kitova, George Goranov, Krasimir Kraev, Maria Kraeva, Lyudmila Kitova

**Affiliations:** 1 First Department of Internal Diseases, Section of Cardiology, Medical Faculty, Medical University of Plovdiv, 3000 Plovdiv, Bulgaria; spas.kitov@mu-plovdiv.bg (S.K.); georgi.goranov@mu-plovdiv.bg (G.G.); kitov@vip.bg (L.K.); 2 Clinic of Cardiology, St. George University Hospital, 4000 Plovdiv, Bulgaria; 3 4th Year Medical Student, Faculty of Medicine, Medical University of Plovdiv, 4000 Plovdiv, Bulgaria; maria_kitova21@abv.bg; 4 Department of Propedeutics of Internal Diseases, Medical Faculty, Medical University of Plovdiv, 3000 Plovdiv, Bulgaria; 5 Department of Otorhinolaryngology, Medical Faculty, Medical University of Plovdiv, 4002 Plovdiv, Bulgaria; kraevamaria93@gmail.com

**Keywords:** alkaptonuria, aortic valve stenosis, transcatheter aortic valve implantation, surgical aortic valve replacement

## Abstract

Introduction: Alkaptonuria is an autosomal-recessive disorder affecting the metabolism of tyrosine and phenylalanine which results in accumulation of homogentisic acid in connective tissues. The joints are most commonly affected, while the most common cardiac damage is aortic valve stenosis. The treatment focuses on reducing the symptoms. Aortic stenosis in alkaptonuria is treated with surgical aortic valve replacement; however, transcatheter aortic valve implantation procedures are increasing in number with excellent outcomes. Case presentation: We report a case of a 67-year-old female with chronic back pain and large-joint arthralgia, who was recently diagnosed with alkaptonuria. She reported a long-known heart murmur and intermittent dark-brown staining of her underwear since childhood. Bilateral dark-brown pigmentation of the sclera and both ear cartilages were visualised. ECG confirmed atrial fibrillation and left ventricular hypertrophy. Cardiac ultrasonography showed severe aortic stenosis, reduced global longitudinal strain and preserved ejection fraction. According to the latest recommendations, the choice between surgical and transcatheter intervention must be based upon careful evaluation of clinical, anatomical and procedural factors by the Heart Team, weighing the risks and benefits of each approach for an individual patient. The advantages and disadvantages of both procedures were explained to the patient. It was emphasised that the genetic disease present has no etiopathogenetic definitive treatment and the pigment may continue to deposit on the biological valve (in transcatheter aortic valve implantation) and less likely on the mechanical valve prosthesis (in Surgical Aortic Valve Replacement), highlighting the fact that in the literature worldwide, there are only single reports of ochronosis and severe aortic stenosis. At this stage of knowledge, it is difficult to give the patient clear guarantees when choosing a methodology for performing a valve correction. Along with the standard therapy the patient underwent transcatheter aortic valve implantation with Boston Scientific prosthesis with a very good post-procedural outcome. Conclusions: There is scarce information on transcatheter aortic valve implantation success rate in patients with alkaptonuria. In the population, transcatheter aortic valve implantation outcome is generally good; however, the individual success in alkaptonuria patients depends on the severity of their heart valve damage and overall health.

## 1. Introduction

Alkaptonuria is a rare autosomal-recessive disorder that affects the metabolism of tyrosine and phenylalanine, resulting in the accumulation of homogentisic acid [HGA] in the body [1,2,3]. HGA binds to fibrillar collagen in the extracellular matrix in the presence of mucopolysaccharides, leading to oxidative stress and tissue damage [3,4].

Alkaptonuria is rare genetic disorder of tyrosine metabolism manifesting with signs of tissue pigmentation, dark urine, and ochronotic arthropathy. Commonly undiscovered by late adulthood, alkaptonuria can manifest as cardiac ochronosis with cardiovascular disorders such as valvulopathy, but rarely coronary artery disease. Being a rare and clinically heterogenous disorder, ochronosis is a serious diagnostic challenge with a dark-stained path to the correct diagnosis. AKU is not life threatening but reduces the quality of life. Early diagnosis and regular follow-up can provide timely treatment of target organ damage and provide a better quality of life.

One of the most frequent sites for pigment deposition is the aortic valve, as well as the intervertebral discs of the lumbar spine, the skin, the ear cartilage and the eyes. Common clinical symptoms include dark urine, ochronotic arthropathy and spondylopathy, which manifest as joint pain. Dark-brown pigmentation of the conjunctiva, cornea and sclera is also observed, although the eyesight is not affected. The renal system effectively secretes HGA; therefore the clinical manifestation takes 3–4 decades.

The disease prognosis depends on the severity of the heart injury as a result of the pigment deposition in the cardiac structures—coronary arteries, cardiac valves and aorta. The most common cardiac pathology in patients with ochronosis is aortic valve stenosis [5].

Diagnosing ochronosis is complex and includes medical history, physical examination, urine and blood laboratory testing and imaging. The concentration of homogentisic acid in body fluids is crucial; however, it can be directly measured by gas chromatography or mass spectrometry analysis. Genetic testing with PCR evaluates HGD mutations. Imaging methods such as X-ray, CT-scan and MRI prove typical changes in the skeletal bones. Arthroscopy and skin biopsy are very informative [6]. Cardiovascular pathology is found on electrocardiography [ECG], echocardiography [EchoCG] and interventional coronarography.

Currently, there is no cure for alkaptonuria—the disease management focuses on reducing symptoms and slowing down the progression. It includes anti-inflammatory medications, pain management and physical therapy. Low tyrosine and phenylalanine diet may also be recommended. Arthroplasty is a common joint damage and is among the most severe manifestations. In patients with alkaptonuria, the treatment of aortic stenosis with SAVR or TAVI requires a careful Heart Team assessment with the same criteria as for patients without alkaptonuria [7,8,9].

## 2. Case Presentation

**Figure 1:**
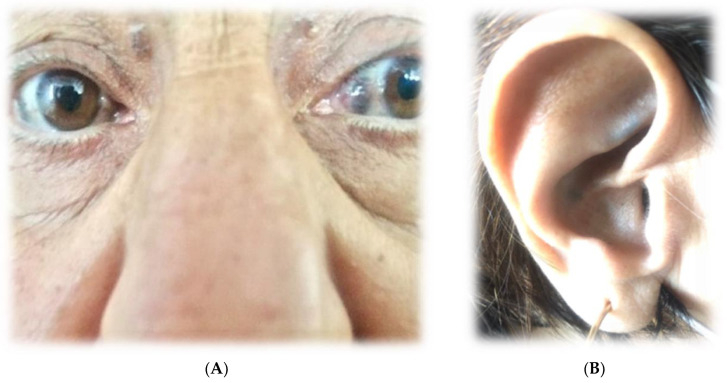
(**A**). Bilateral dark-brown pigmentation of the sclera. (**B**). Bluish-black discoloration of both ear cartilages.

We report a case of a 67-year-old female who complained of dyspnea, retrosternal discomfort and fatigue during mild physical activity, as well as orthopnea. She also reported edema of the lower parts of both legs and chronic back and joint pain for more than 30 years. With relation to the joint complaints, the patient first came to the Clinic of Rheumatology a few months prior, where the diagnosis of seronegative spondyloarthritis with ochronosis was made. The following findings confirm the diagnosis:

1. There was dark staining of her underwear.
2. Upon physical examination, there was bilateral dark-brown pigmentation of the sclera [Figure 1A] and bluish-black discoloration of both ear cartilages [Figure 1B]. She first noticed dark pigmentation in her eyes at the age of 30, but this had raised no concern since her father had similar changes at approximately the same age.
3. The physical examination found: the patient’s range of motion in the spine, shoulders and knees was limited. In addition, muffled heart sounds and a rough systolic murmur propagating towards the carotid arteries with a punctum maximum on the aortic valve were auscultated.
4. Based on the history, physical examination and instrumental studies, ochronosis was considered in the differential diagnostic plan. The patient was referred by a rheumatologist to HGA concentration testing in her blood and urine, and it was significantly elevated—it was above the upper limit of the reference range—1.54 g/24 h. Therefore, the diagnosis of alkaptonuria was confirmed.

## 3. Cardiac Findings

The ECG confirmed atrial fibrillation with left ventricular hypertrophy. EchoCG revealed left ventricular symmetrical concentric hypertrophy. Pathological aortic valve pressure gradients were observed—the maximum one—110 mmHg and average gradient—69 mmHg.

The heart contractility was also decreased due to the persistent higher afterload. A decrease in left ventricular ejection fraction was found in the grey area [48%], indicating chronic moderate heart decompensation. The measured mean left ventricular longitudinal strain was also reduced [−10%].

**Figure 2:**
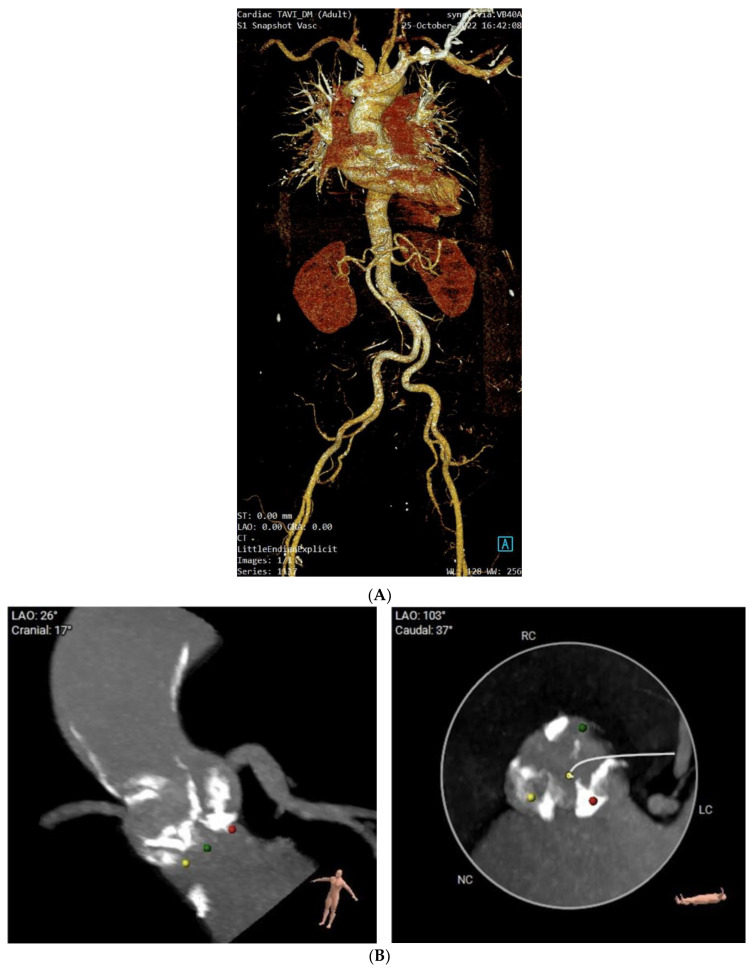
(**A**). Computed tomography angiography. (**B**). Moderate aortic valve calcifications.

In the course of the diagnostic algorithm, an invasive examination was performed. The valve assessment confirmed a high transaortic gradient and severe aortic stenosis, respectively. The mitral valve presented with low-grade regurgitation. Selective coronary angiography showed normal coronary anatomy. Computed tomography angiography did not show a porcelain aorta [Figure 2A] nor severe aortic valve calcification [Figure 2B].

## 4. Patient Management

At the next stage, the patient was presented to the heart team to determine the therapeutic approach. The treatment variants were minimally invasive endoscopic aortic valve replacement via right anterior minithoracotomy or invasive treatment with transcatheter aortic valve implantation. All aspects (clinical characteristics, anatomical and technical aspects and additional conditions) that the expert should take into account when deciding between Surgical Aortic Valve Replacement [SAVR] and Transcatheter Aortic Valve Implantation [TAVI] in patients with severe aortic stenosis were discussed. This is in accordance with the clinical guidelines of the European Society of Cardiology and the European Society of Cardiothoracic Surgery [10]. The European System for Cardiac Operative Risk Evaluation [EuroSCORE] II predicts risk of in-hospital mortality after cardiac surgery was low risk—1.9%. The Society of Thoracic Surgeons Predicted Risk of Mortality [STS-PROM] score—8.9%.

In terms of specific surgical groups, EuroSCORE II was most useful for estimating risk in patients who had either stand-alone Coronary Artery Bypass Grafting [CABG] or isolated mitral valve surgery, whereas the STS score was most useful for patients who had either AVR only or CABG with concomitant valve surgery. The STS score in our patient was above 8%, which is considered high surgical risk; however, according to EuroSCORE II, it is a low surgical risk.

According to the latest recommendations, the choice between surgical and transcatheter intervention must be based upon careful evaluation of clinical, anatomical and procedural factors by the Heart Team, weighing the risks and benefits of each approach for an individual patient. The individual characteristics of our patient are presented in Table 1.

In terms of clinical characteristics alone, SAVR outperforms the TAVI procedure. In terms of anatomical and technical aspects, the two approaches are equal. The TAVI procedure is superior to SAVR in cardiac conditions in addition to aortic stenosis that require concomitant intervention. The Heart Team recommendation should be discussed with the patient who can then make an informed treatment choice. The advantages and disadvantages of both procedures were explained to the patient. It was emphasised that the genetic disease present has no etiopathogenetic definitive treatment and the pigment may continue to deposit on the biological valve /in TAVI/ and less likely on the mechanical valve prosthesis /in SAVR/, highlighting the fact that in the literature worldwide, there are only single reports of ochronosis and severe aortic stenosis [11,12,13,14]. At this stage of knowledge, it is difficult to give the patient clear guarantees when choosing a methodology for performing a valve correction. The definite refusal of operation on the side of the patient in the presence of the available anatomical and technically equal positions of the two procedures led to the decision to perform the TAVI procedure. Standard protocol was applied. She subsequently underwent successful transfemoral TAVR with a 23 mm Acurate Neo2 (Boston Scientific). In this case, the Accurate Neo2 (Boston Scientific) self-expandable valve was chosen due to the presence of a tortuous ilio-femoral tract. Self-expandable valves are typically preferred for patients with smaller or more tortuous vessels due to their lower delivery system profile. Another important factor was the height of the left coronary artery (LCA), which measured 9.7 mm. The Accurate Neo 2 valve features extra-large open cells, which enhance access to the coronary arteries, making it easier to manage any potential complications. Additionally, the low risk of interference with the heart’s conduction system and the fact that rapid ventricular pacing is not needed during the valve’s deployment were also key considerations in selecting this valve.

Transesophageal echocardiography performed after valve deployment revealed a trace paravalvular leak with peak and mean gradients of 18.5 and 7.5 mm Hg, respectively. The procedure was carried out with no complications, and the patient was discharged in improved general condition. She was discharged 2 days following the procedure. After TAVI, the patient continued therapy with beta-blocker, angiotensin-converting enzyme inhibitors, anticoagulant, sodium-glucose cotransporter 2 inhibitors and aldosterone antagonist. The choice of medical therapy was based on the initial diagnosis of heart failure with mildly reduced ejection fraction and permanent atrial fibrillation.

In the presented case, we did not use near infrared spectroscopy for monitoring cerebral oximetry, which is recommended in these cases [13] due to lack of the device. The absence of severe aortic valve calcification gave us the courage to perform the procedure [13].

**Figure 3:**
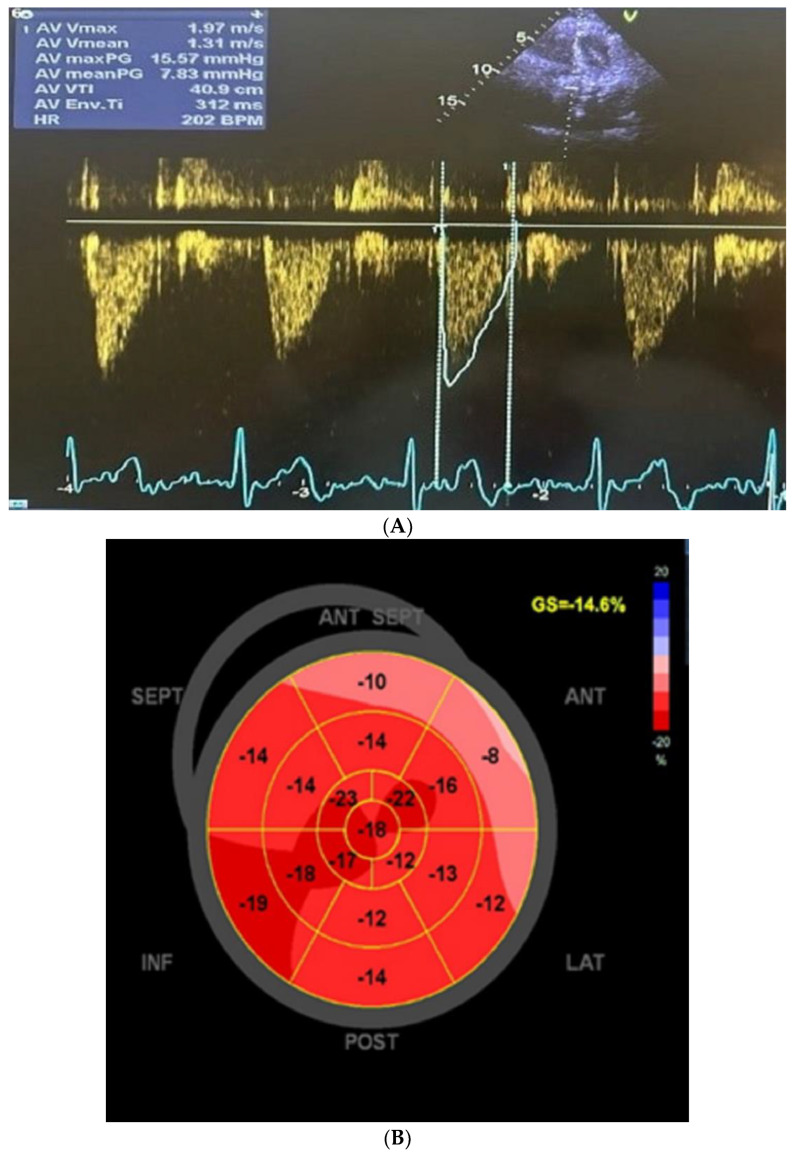
(**A**). Aortic valve gradient two and a half years after the procedure. (**B**). The bull’s-eye plot polar map representing the global longitudinal strain (GLS) of the left ventricle.

The patient was monitored every 3 months for 2.5 years, and the ejection fraction has normalised; the valve had a normal gradient. However, the longitudinal strain is still decreased due to irreversible heart damage [Figure 3A,B].

## 5. Discussion and Conclusions

There have been increasing reports of cardiovascular ochronosis [5,6,7,8]. It is well accepted that ochronosis is associated with aortic valve stenosis, but mitral and pulmonary valves can be affected, and multiple valves may be involved. The presented clinical case confirms these data only with respect to the aortic valve. The pigment is usually most marked at the valve cusp or at the leaflet base extending into the valve annulus. The pathogenesis of aortic valve calcification and aortic stenosis is probably related to the extensive extracellular deposits of ochronotic pigment. Gaines et al. suggest that the anatomical derangement of the valves is caused by the degeneration of pigment-laden fibrocytes, which leads to progressive calcification, and by valve substance fibrosis [1], but considering the disease nature and theories proposed, the mechanical valve seems a better choice, irrespective of the age [15,16]. There is no consensus on the choice of the valve in ALK patients. There is a lack of evidence to prove the benefit of either the mechanical or bioprosthetic valves in these patients. Considering the pathophysiology of ALK, there are possibilities of HGA deposition and calcification over the biological tissue in the bioprosthetic [14].

Regarding the choice of a mechanical or bioprosthetic valve in patients with alkaptonuria-associated aortic valve disease, prosthetic valves were used in most cases, in line with patient preference and the age-specific recommendations in guidelines. Treatment for alkaptonuria-associated AS has mostly involved surgical aortic valve replacement, and there have been few reports of successful TAVR [12,17].

Thalur and colleagues [18] reported that they used a mechanical valve because bioprosthetic valves might further the calcification, given the degenerative mechanism of native valves in alkaptonuria. However, there has been no report of structural valve deterioration due to bioprosthetic valve calcification used in SAVR in alkaptonuria patients.

Capuano et al. [19] report on SAVR performed in a patient with alkaptonuria complicated by Heyde’s syndrome, a condition with gastrointestinal bleeding due to angiodysplasia in the presence of severe AS. Given the potential postoperative bleeding, they chose a bioprosthetic valve.

Nitisinone, which is used for type I tyrosinemia, has been reported to be effective for the treatment of alkaptonuria since it lowers plasma HGA levels [1,20]. However, nitisinone has not been confirmed to prevent the progression of aortic valve disease [20], and there have been no reports showing suppression of bioprosthetic valve calcification.

It is unclear whether biological prosthetic valves are exposed to the same process of HGA deposition and calcification, which would affect prosthesis longevity [11].

Aortic stenosis in our patient was found together with ochronosis, i.e., aged 67 years, in similarity to other cases in the literature [21]. This fact shows that ochronosis changes the quality of life, but not its duration. The report of Thakur et al. is essential in that in the case of ochronosis, around the age of 40, screening with echocardiography and CT angiography should be carried out [18].

To date, all reported cases of aortic involvement in ochronosis have presented with a stenotic phenomenon, i.e., aortic stenosis, as is our case. There is only one reported case of aortic regurgitation, reported by Yoshikai and colleagues [22]. This fact necessitates the need to optimise the treatment approach in the developing aortic stenosis, i.e., the need for clarity regarding the advantages of TAVI or SAVR in ochronosis.

Minimally invasive surgical aortic valve replacement was discussed in the patient’s case as possible surgical treatment with a lower complication risk. There are scarce data in the literature for successful minimally invasive access in ochronosis [16]. This method is a good alternative to TAVI; however, ochronosis hides the risks of impaired surgical incision healing. Furthermore, the patient refused the option.

Regarding the patient presented in the article, the choice of TAVI is based on a complex clinical, anatomical, technical evaluation, absence of accompanying heart valve and vascular pathology, as well as the patient’s preference. The advantages of TAVI over open-heart surgery are undeniable—less invasive, lower perioperative risk and quicker postoperative recovery. There is limited information on the success rate of TAVI in patients with alkaptonuria [11,12,13,14]. TAVI was chosen for this patient due to increased cases of suture dehiscence in ochronosis patients, resulting from connective tissue insufficiency caused by ochronotic pigment deposition. Another reason for this decision is the more difficult recovery period after SAVR, as patients with this disorder usually have arthropathy, which disables them from movement, crucial for rehabilitation after SAVR. In addition, the absence of accompanying coronary artery disease is a very important argument for choosing TAVI. The idea that in the case of stenosis of the bioprosthesis during TAVI the valve-in-valve method can be performed if necessary and is still less expensive than open surgery came up.

Coronary artery disease is commonly present in patients with aortic stenosis because the process of proliferative and inflammatory changes with lipid accumulation and infiltration of macrophages into aortic valve leaflets is similar to that of atherosclerosis [4,7]. Conversely, it has also been concluded that HGA-oxidized depositions have effects on the endothelial surface that cause dysfunction and possible development of coronary artery disease. Such vascular abnormalities have been previously described in several cases of cardiovascular ochronosis [3,4,5]. In contrast to the literature data, the present clinical case patient did not present with concomitant CHD. This fact provides an additional very important reason for considering a TAVI procedure in these patients.

At follow-up in months 1, 3, 6 and 12, transthoracic echocardiography revealed a well-seated valve with mild paravalvular aortic regurgitation, peak and mean pressure gradients of 15 and 7 mm Hg, respectively, an aortic valve area of 1.8 cm2 and an ejection fraction of 55%. The patient reported significant improvement in symptoms after the procedure. The inclusion of renin-angiotensin aldosterone system (RAAS) inhibitors in the therapeutic strategy (she had not received TAVI before) is related to the need for reverse remodelling in hypertensive patients with severe aortic stenosis. According to data from the EffecTAVI registry, in hypertensive patients with severe aortic stenosis, RAAS treatment was found to be independently associated with a lower risk of 2-year cardiovascular mortality. The inhibitory effect of RAAS on hypertrophy and fibrosis is a possible explanation for the link between RAAS and mortality. This is even more relevant for patients with alkaptonuria and may explain the good clinical course of the patient for the past 2.5 years [23]. The presence of atrial fibrillation could mainly be explained as secondary to the stenotic changes in the aortic valve with left ventricular pressure overload and subsequent left atrial remodelling and enlargement.

In the population, TAVI outcomes are generally good; however, the individual prognosis in alkaptonuria patients depends on the severity of their heart valve damage and overall health [15,16]. A follow-up study is required to develop a fixed protocol about the valve choice.

There are three clinical messages from this clinical case:

1. The need for inclusion of RAAS inhibitors in hypertensive patients with severe aortic stenosis (before and after TAVI) is important for myocardial remodelling and clinical course.
2. In alkaptonuria, the choice of intervention for severe aortic stenosis should be based on the individual specifics of the patient and follow the standard criteria for risk assessment by the Heart Team.
3. TAVI is a reliable choice in alkaptonuria-associated severe aortic stenosis.

## 6. Patient Perspective

In conclusion, the present case adds further information to the prevailing opinion that therapeutic behaviour in alkaptonuria-associated aortic stenosis should be individualised to the specific case. Two-and-a-half-year follow-up is a good starting point for TAVI in alkaptonuria-associated aortic stenosis. There are little data in the literature on longer follow-up periods in this type of patients. Observation of the patient continues. The TAVI procedure is constantly evolving, and the optimal option for patients with ochronosis should probably be found in the future [24].

## Figures and Tables

**Table 1:** Clinical, anatomical and procedural factors in favour of Surgical Aortic Valve Replacement or Transcatheter Aortic Valve Implantation.

	In Favour of TAVI	In Favour of SAVR
Clinical Characteristics		
STS-PROM/EuroSCORE II < 4% [logistic EuroSCORE I < 10%]		
STS-PROM/EuroSCORE II > 4% 4.68% [logistic EuroSCORE I > 10%]		1 8.9/1.9%
Presence of significant comorbidity [Inadequately recorded in the scores]		1
Age below 75 years		1
Age above 75 years		
Previous cardiac surgery		1
Frailty		1
Limited mobility and conditions that may affect the rehabilitation process following the procedure		1
Suspected endocarditis	1	
Total	2	5
**Anatomical and technical aspects**	**In favour of TAVI**	**In favour of SAVR**
Appropriate transfemoral access for TAVI	1	
Inappropriate [different] access for TAVI	1	
Thoracal radiotherapy consequences		1
Porcelain aorta		1
Presence of operating by-pass with sternotomy risk		1
Expected prosthesis–patient mismatch		1
Pronounced thoracic deformity or scoliosis		1
Short distance between coronary ostia and aortic annulus		1
Aortic annulus size out of range for TAVI	1	
Aortic root morphology unfavourable for TAVI	1	
Morphology [bicuspidia, calcification type and degree unfavourable for TAVI]	1	
Thrombus presence in the aorta or LC	1	
Total	6	6
**Cardiac conditions apart from aortic stenosis requiring concomitant intervention**	**In favour of TAVI**	**In favour of SAVR**
Significant coronary disease requiring revascularization through CAB	1	
Significant primary mitral valve disease requiring surgical treatment	1	
Significant tricuspid valve disease	1	
Ascending aortic aneurism	1	
Septal hypertrophy requiring myomectomy	1	
Total	5	

## Data Availability

Dataset available on request from the authors

## Abbreviations

The following abbreviations are used in this manuscript:

**AKU**	Alkaptonuria
**PCR**	Polymerase Chain Reaction
**EchoCG**	Echocardiography
**AS**	Aortic Stenosis
**SAVR**	Surgical Aortic Valve Replacemen
**TAVI**	Transcatheter Aortic Valve Implantation
